# Evaluating Physiological MRI Parameters in Patients with Brain Metastases Undergoing Stereotactic Radiosurgery—A Preliminary Analysis and Case Report

**DOI:** 10.3390/cancers15174298

**Published:** 2023-08-28

**Authors:** Eva E. van Grinsven, Jordi de Leeuw, Jeroen C. W. Siero, Joost J. C. Verhoeff, Martine J. E. van Zandvoort, Junghun Cho, Marielle E. P. Philippens, Alex A. Bhogal

**Affiliations:** 1 Department of Neurology & Neurosurgery, University Medical Center Utrecht Brain Center, Utrecht University, 3584 CX Utrecht, The Netherlands; 2 Department of Radiology, Center for Image Sciences, University Medical Center Utrecht, 3584 CX Utrecht, The Netherlands; jdl1997@live.nl (J.d.L.); a.bhogal@umcutrecht.nl (A.A.B.); 3 Spinoza Center for Neuroimaging, 1105 BK Amsterdam, The Netherlands; 4 Department of Radiation Oncology, University Medical Center Utrecht, 3584 CX Utrecht, The Netherlandsm.philippens@umcutrecht.nl (M.E.P.P.); 5 Department of Experimental Psychology, Helmholtz Institute, Utrecht University, 3584 CS Utrecht, The Netherlands; 6 Department of Biomedical Engineering, SUNY Buffalo, Buffalo, NY 14228, USA; jcho34@buffalo.edu

**Keywords:** brain metastases, cerebral blood flow, cerebral metabolic rate of oxygen, cerebrovascular reactivity, cognition, oxygen extraction fraction, radiotherapy, stereotactic radiosurgery

## Abstract

**Simple Summary:**

Brain metastases affect up to thirty percent of the adult cancer population. One of the treatment options is stereotactic radiosurgery (SRS). To better understand SRS-related side-effects, we used advanced imaging techniques to study both the vascular and metabolic reserve capacity of the brain in nine patients with brain metastases before and three months after SRS. We observed larger declines in both vascular and metabolic markers in healthy brain regions that received a higher dose of SRS, but also saw substantial variation within- and between individuals. Our case analysis showed that the markers used in this study were able to pick up differences between patients who had either tumor growth or tumor shrinkage three months after SRS. These preliminary findings suggest there are no immediate detrimental effects on healthy brain tissue following SRS, but larger groups with a longer follow-up are needed to investigate potential late side-effects.

**Abstract:**

Brain metastases occur in ten to thirty percent of the adult cancer population. Treatment consists of different (palliative) options, including stereotactic radiosurgery (SRS). Sensitive MRI biomarkers are needed to better understand radiotherapy-related effects on cerebral physiology and the subsequent effects on neurocognitive functioning. In the current study, we used physiological imaging techniques to assess cerebral blood flow (CBF), oxygen extraction fraction (OEF), cerebral metabolic rate of oxygen (CMRO_2_) and cerebrovascular reactivity (CVR) before and three months after SRS in nine patients with brain metastases. The results showed improvement in OEF, CBF and CMRO_2_ within brain tissue that recovered from edema (all *p* ≤ 0.04), while CVR remained impacted. We observed a global post-radiotherapy increase in CBF in healthy-appearing brain tissue (*p* = 0.02). A repeated measures correlation analysis showed larger reductions within regions exposed to higher radiotherapy doses in CBF (*r_rm_* = −0.286, *p* < 0.001), CMRO_2_ (*r_rm_* = −0.254, *p* < 0.001), and CVR (*r_rm_* = −0.346, *p* < 0.001), but not in OEF (*r_rm_* = −0.004, *p* = 0.954). Case analyses illustrated the impact of brain metastases progression on the post-radiotherapy changes in both physiological MRI measures and cognitive performance. Our preliminary findings suggest no radiotherapy effects on physiological parameters occurred in healthy-appearing brain tissue within 3-months post-radiotherapy. Nevertheless, as radiotherapy can have late side effects, larger patient samples allowing meaningful grouping of patients and longer follow-ups are needed.

## 1. Introduction

Approximately twenty percent of the basal metabolic rate of the body is consumed by the human brain, making it the most energy demanding organ [1,2]. The primary sources of energy for the brain include oxygen and glucose, which are delivered by arterial blood. To ensure the adequate delivery of nutrients necessary for homeostasis, autoregulatory functions serve to maintain stable cerebral blood flow (CBF) in response to changes in perfusion pressure or other hemodynamic events [2,3]. However, this autoregulatory system can be disturbed by the presence of brain metastases.

Brain metastases represent a distressing aspect of cancer progression and occur in ten to thirty percent of the adult cancer population [4,5]. To metastasize to the brain, cancer cells sequentially complete a series of processes (e.g., penetration of the blood–brain barrier) [6] that may lead to alterations in the surrounding metabolic and vascular microenvironment [7,8,9,10]. Moreover, both primary brain tumors and brain metastases are frequently surrounded by vasogenic edema, which is the result of local blood–brain barrier disruptions, allowing protein-rich fluid to accumulate in the extracellular space [11]. A previous study has reported a lower fractional extraction of oxygen in edema surrounding diffuse gliomas and lower regional blood flow in edema surrounding brain metastases [12]. Moreover, impaired cerebrovascular reactivity (CVR) has been observed within edema surrounding both diffuse gliomas [13] and brain metastases [14], which could reflect a local pressure effect restricting the ability of vessels to dilate and maintain adequate perfusion. Treatment and management of edema is aimed at reducing swelling and alleviating symptoms. However, it is unknown whether the metabolic and vascular reserves within these regions also recover once edema subsides.

Radiotherapy is the cornerstone of (palliative) treatment for brain metastases, in combination with surgery, chemotherapy or immunotherapy [15]. Unfortunately, brain radiation can also damage surrounding healthy tissue, as shown by largely reduced vessel density in the brain after fractionated radiotherapy in rats [16]. Likewise, studies have found vascular damage resulting in reduced blood perfusion in brain areas that received at least 10–15 Gy [17]. Other studies have even shown reduced CBF at radiation doses below 10 Gy [18]. Research on metabolic changes after radiotherapy is rather limited. Hypothetically, radiation damage to vessels could reduce the ability to regulate CBF, leading to an increased oxygen extraction fraction (OEF) in order to maintain a sufficient cerebral metabolic rate of oxygen (CMRO_2_). On the other hand, the irradiation of healthy brain tissue can also cause cell damage [19], possibly resulting in a lowered metabolism and need for oxygen. Conclusively, the various metabolic and vascular changes that can occur after radiotherapy highlight the complexity of radiation-induced damage.

These complex and multifaceted changes in the brain may partially explain the large individual differences in radiation-induced cognitive decline experienced by patients with brain metastases [20]. Non-invasive, sensitive MRI biomarkers are needed to better understand radiotherapy-related changes in the brain and its subsequent effect on neurocognitive functioning. With this in mind, the main aim of the current study was to assess the added value of using physiological imaging techniques to assess CVR, CBF, OEF and CMRO_2_ in both healthy-appearing tissue and malignant tissue before and after stereotactic radiosurgery (SRS) in patients with brain metastases. Acquiring both OEF and CBF in the same scan session can provide insight as to whether changes in brain oxygen metabolism (i.e., CMRO_2_) occur due to abundant or insufficient blood supply or because the tissue itself has (partly) lost the ability to extract and consume oxygen. The addition of a CVR measurement can give insight into the regulatory state of the tissue at baseline. As a secondary analysis, we investigated cognitive changes after radiotherapy in relation to changes in these physiological MRI parameters on a case-by-case basis. This preliminary study aims to establish a foundational framework for advancing our understanding of radiation-induced brain damage and its relation to cognitive functioning, thereby providing a basis to generate novel hypotheses to guide future research.

## 2. Materials and Methods

### 2.1. Study Set-Up and Population

MRI datasets were acquired between October 2022 and February 2023 for the ongoing Assessing and Predicting Radiation Influence on Cognitive Outcome using the cerebrovascular stress Test (APRICOT) study. Participation consists of an elaborate neurocognitive assessment (NCA) and MRI before radiotherapy and approximately three months after radiotherapy. The study population consists of adult patients (≥18 years) with either radiographic and/or histologic proof of metastatic disease in the brain that were referred to the radiotherapy department of the UMC Utrecht for radiotherapy of the brain metastases. Specific in- and exclusion criteria for participation in the APRICOT study are listed in Appendix A. The study was performed in accordance with the Declaration of Helsinki [21] and the UMCU institutional ethical review approved the study (METC# 18-747). Written informed consent was obtained from all participants prior to participation.

### 2.2. Data Acquisition

#### 2.2.1. Imaging Protocol

The participants were scanned on a 3 Tesla MRI scanner (Achieva, Philips Medical Systems, Best, The Netherlands) using a 32-channel receive coil. The same scanning protocol was used as described before [14]. To acquire the CVR measurements, whole-brain multi-slice single-shot gradient-echo EPI BOLD images (TR = 1050 ms, TE = 30 ms, flip angle: 65°, voxel size: 2.292 × 2.292 × 2.5 mm^3^, acquisition matrix: 96 × 96 × 51, 1000 dynamics, multi-band factor = 3, SENSE factor = 1.8) were acquired through a computer-controlled hypercapnic breathing protocol (described below). An additional dataset pair was acquired for EPI phase (distortion) correction. Perfusion data were acquired using a multi-delay ASL sequence. A whole volume was acquired at 4 post-labeling delays (660, 1325, 1989, 2654 ms), using a pCASL Look-Locker multi-slice EPI read-out (total scan time = 240 s, labelling train duration = 1650 ms, TR = 5 s, TE = 12 ms, flip angle: 25°, acquired resolution: 3 × 3 × 7 mm^3^, acquisition matrix: 80 × 80 × 17, 23 volumes of label-control pairs, SENSE factor = 2, 2 background suppression pulses). The first volume contained the calibration (M0) images for each post-label delay where the labelling, saturation pulses and background suppression were turned off. No breathing challenges were performed during the ASL imaging. The ASL was planned using a fast phase contrast angiography scan, with the labeling plane carefully placed perpendicular to the internal carotid arteries and vertebral arteries. The whole brain SWI was acquired using a multi-echo gradient-echo (ME-GRE) sequence (TE1 = 8.5 ms, TE2 = 17.5 ms, TE3 = 26.5 ms, TE4 = 35.5 ms, TE5 = 44.5 ms, echo spacing: 8 ms, TR: 50 ms, flip angle: 17°, voxels size: 0.342 × 0.342 × 2 mm^3^, acquisition matrix: 384 × 383 × 63). Additionally, a 3D T1-weighted magnetization-prepared rapid gradient-echo imaging (MPRAGE) sequence (TR = 8 ms, TE = 3.25 ms, flip angle: 10°, isotropic resolution: 1 mm, acquisition matrix: 240 × 240 × 180) and a 3D T2-weighted FLAIR sequence (TR: 4800 ms, TE: 240 ms, TI = 1650 ms, flip angle: 90°, isotropic resolution: 1 mm, acquisition matrix: 256 × 256 × 182) were acquired for anatomical reference.

#### 2.2.2. Clinical CT and MRI Acquisition

**Figure 1:**
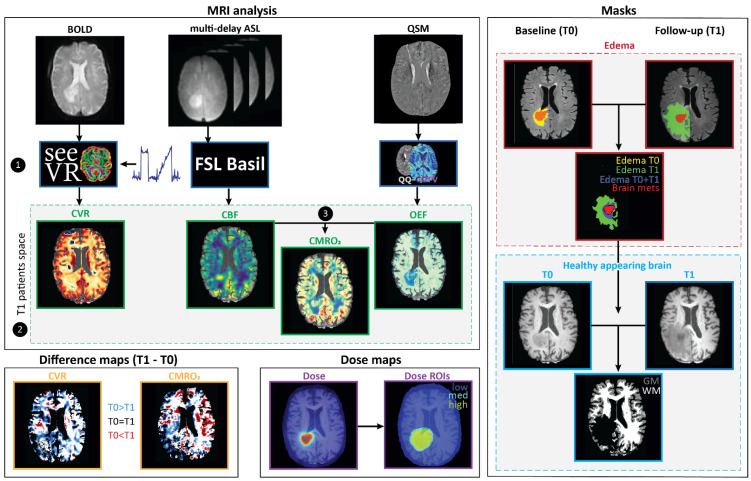
Data analysis steps for the MRI data. MRI analysis was performed for the BOLD, ASL and QSM data. (1) CVR maps were generated with the SeeVR toolbox using the BOLD time series and PetCO_2_ traces, quantitative CBF maps were generated based on the multi-delay ASL data using the ClinicalASL toolbox and FSL BASIL, and OEF maps were generated from the QSM data with the QQ-CTV software package. (2) All MRI maps were transformed to the baseline T1 patient space. (3) The CMRO_2_ map was generated by multiplying the CBF map with the OEF map. Multiple masks were generated. Edema masks were made based on the T2FLAIR hyper intensities for both baseline (T0) and follow-up (T1). From this, new masks including regions only exhibiting edema at baseline (edema T0), regions only exhibiting edema at follow-up (edema T1) and regions exhibiting edema at both time points (edema T0 + T1) were created. The brain metastases were excluded from the edema regions. Next, the edema and brain metastases masks were excluded from the whole-brain mask to create a mask with healthy-appearing brain tissue at both time points. This healthy-appearing brain tissue was subdivided into grey matter (GM) and white matter (WM). Difference maps subtracting the follow-up (T1) data from the baseline (T0) were calculated for CVR and CMRO_2_. Positive values represent an increase in either CVR or CMRO_2_ at follow-up (T0 < T1) and negative values represent a decrease in either CVR or CMRO_2_ at follow-up (T0 > T1). The subtraction process was restricted to pixels that contained values in both the pre- and post-radiotherapy images. Images were masked to exclude CSF. Finally, dose maps were extracted with one map showing the total delivered dose in EQD2 and the other map dividing the dose into ROIs with low (<10 Gy), medium (10–15 Gy) and high (>15 Gy) doses, as described above.

CT and MRI scans were acquired as part of clinical care as usual, 1 to 5 days before receiving SRS. CT scans were acquired on a Brilliance Big bore 22 scanner (Philips Medical Systems, Best, The Netherlands) with a tube potential of 120 kVp, matrix size of 512 × 512 and inplane slice thickness of 1 mm. The participants were scanned on a 1.5 Tesla MRI scanner (Ingenia, Philips Medical Systems, Best, The Netherlands) using a 15-channel receive coil. A 3D SPGR sequence after injection of 0.1 mL gadovist/kg was performed (TR = 7.6 ms, TE = 3.4 ms, flip angle: 8°, isotropic resolution: 1 mm, acquisition matrix: 232 × 232 × 170). The clinician used this clinically acquired MRI, registered to the CT, to plan the radiotherapy and delineate the so-called gross tumor volume (GTV; i.e., brain metastases). The prescribed dose was recalculated into equivalent total doses per 2-Gy fractions (EQD2) to aid comparability between and generalizability to different radiation schemes. The CT, dose maps, planned target volume (PTV) and GTV were extracted for each patient. For the current analysis, the GTV was divided into newly treated brain metastases, resection cavities of brain metastases and previously irradiated brain metastases. Analyses were only performed on newly treated brain metastases. If a patient had multiple brain metastases, averages were calculated from all voxels corresponding to the multiple brain metastases. Dose maps were subdivided into regions of interests (ROIs) with low (<10 Gy), medium (10–15 Gy) and high (>15 Gy) doses, following previous research on vascular damage described at radiotherapy doses higher than 10–15 Gy [17] (Figure 1).

#### 2.2.3. Breathing Protocol

**Figure A1:**
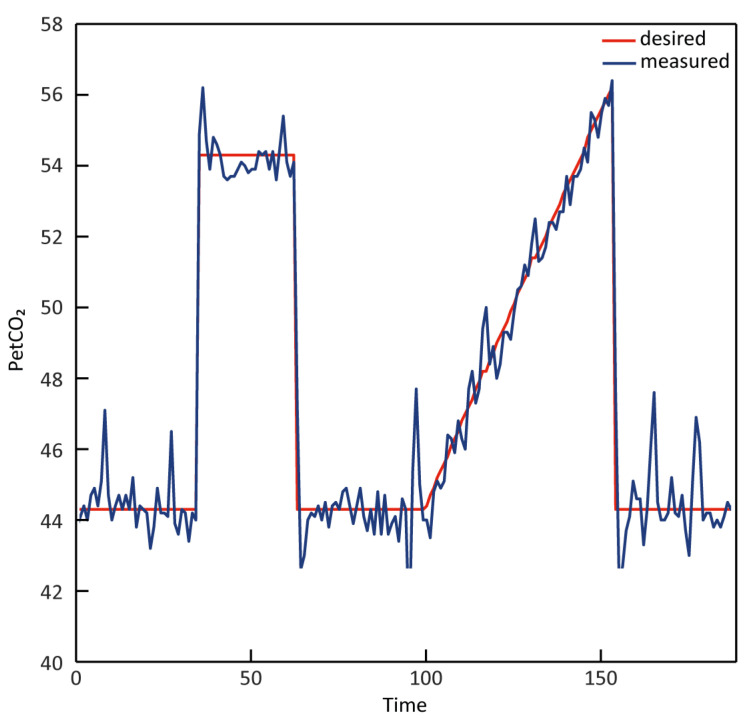
CO_2_ breathing trace of a representative subject during the breathing challenge. The blue line indicates the actual measured PetCO_2_ values, while the red line indicates the desired values based on the protocol.

Hypercapnic stimuli were delivered using a computer-controlled gas blender and sequential delivery system (RespirAct, Thornhill Research Institute, Toronto, Canada). The breathing mask was sealed to the patients’ faces using transparent dressings (Tegaderm, 3M, St. Paul, MN, USA) to acquire an air tight seal. Before starting the breathing challenges inside the MRI scanner, patients performed a test round with a CO_2_ challenge which is similar to the CO_2_ block given during the protocol. Only after successful completion of the test round were the breathing challenges inside the scanner performed (see Figure A1). The breathing challenges started with a 5-min baseline period, followed by a block-shaped increase of end-tidal CO_2_ (PetCO_2_) 10 mmHg relative to a patient’s baseline for 90 s. After this so-called CO_2_-block, the PetCO_2_ values returned to baseline values for 120 s, followed by a PetCO_2_ ramp increase of 12 mmHg relative to the patient’s baseline for 180 s, after which the patient was returned to baseline for 90 s.

#### 2.2.4. Neurocognitive Assessment

An extensive battery of neuropsychological tests was used to assess cognitive performance both before and three months after SRS. All tests are internationally widely used, standardized psychometric instruments for assessing neurocognitive deficits in the major neurocognitive domains (see Table A1) [20,22]. Each neuropsychological test was scored according to standardized scoring criteria. Individual change in neurocognitive performance post-radiotherapy was assessed and classified using the reliable change index (RCI) as formulated by Jacobson and Truax [23,24]. RCI values ≥ 1.645 present improvement, ≤−1.645 present decline and values not exceeding ±1.645 indicate stable cognitive performance on the task. Change in neurocognitive performance per domain was defined as improved if at least one task within that domain showed improvement, as declined if at least one task showed a decline, as mixed if at least one task indicated improvement and one task indicated decline and as stable when all tasks within that domain showed stable performance.

### 2.3. Pre-Processing

Pre-processing steps were performed using the Oxford Centre for Functional MRI of the BRAIN (FMRIB) Software Library (FSL—version 6.0) [25]. First, brain extraction was performed on the T1 and T2 FLAIR images using BET [26]. Tissue segmentation into grey matter (GM), white matter (WM) and cerebrospinal fluid (CSF) was performed on the T1 images using the FSL Automated Segmentation Tool (FAST) [27]. Additionally, an edema mask was created based on the T1 and T2FLAIR images using the lesion growth algorithm as implemented in the Lesion Segmentation Tool (LST, https://www.statistical-modelling.de/lst.html, accessed on 28 June 2021) for SPM [28]. Based on previous experience, the initial threshold was set at 0.14. The resulting edema mask was manually adapted to eliminate any false positives or false negatives from the LST edema mask.

BOLD data were motion-corrected (MCFLIRT) [26], corrected for geometric distortion using TOPUP [29,30] and linear spatial co-registered to the T1 images using the ‘epi_reg’ function [26,31]. QSM maps were calculated from the raw phase and magnitude data of the ME-GRE images using the MEDI toolbox [32]. The local field was estimated by the projection onto dipole field (PDF) method [33], and susceptibility values were computed by Morphology Enabled Dipole Inversion (MEDI) [34].

### 2.4. Data Analysis

#### 2.4.1. MRI Analysis

CVR maps were derived using the open-source seeVR toolbox (Figure 1) [35]. In brief, in order to remove the signal contribution from large veins that might overshadow tissue responses, a modified whole-brain mask was generated using the ‘remLV.m’ function of the seeVR toolbox [35]. For this, a temporal noise-to-signal (tNSR) map was calculated by taking the inverse of the temporal signal-to-noise (tSNR) map. Next, voxels showing values higher than the 98th percentile tNSR value were removed from the original whole brain-mask to remove the large veins. This modified mask was then used in subsequent analyses [35]. Next, a manual bulk alignment was performed between the PetCO_2_ and average GM time series to minimize alignment errors that can occur when using automated correlation methods. Thereby, any bulk delays between the end-tidal gas measurements at the lungs and the BOLD signal responses in the brain were accounted for. Residual motion signals with a correlation higher than 0.3 with the GM time series, along with a linear drift term were regressed out using a general linear model. The BOLD data were then temporally de-noised using a wavelet-based approach [36]. A linear regression was performed between the bulk-aligned PetCO_2_ trace with each processed BOLD voxel time series. The slope of this linear regression was taken as the CVR (%ΔBOLD/mmHg PetCO_2_).

The multi-delay ASL was processed using the open-source ClinicalASL toolbox [37] and FSL BASIL for quantitative CBF maps [38]. As described before [14], a T1-weighted image was generated based on the M0 images from each PLD, which was used for subsequent image registration and outlier removal. Outlier removal was performed based on the SCORE method by Duloi et al. (Figure 1) [39].

ME-GRE data were used to generate OEF maps using the integrative QQ approach of Cho et al. (Figure 1), with an unsupervised machine learning algorithm, temporal clustering, tissue composition and total variation (CCTV) [40,41,42,43,44]. QQ estimates OEF maps based on the QSM processing of the ME-GRE phase and qBOLD modeling of ME-GRE magnitude data. The robustness of QQ against noise was improved substantially by using CCTV [45], which groups voxels with similar signal evolution and the same tissue type (GM/WM/CSF) into a cluster and assumes that the voxels have similar model parameters including OEF.

All MRIs were registered to the T1 image acquired at baseline using FMRIB’s Linear Image Registration Tool (FLIRT; Figure 1) [26,31]. Next, CMRO_2_ maps were generated by combing the OEF and CBF data according to the following equation:

(1) $${CMRO}_{2}=OEF\cdot CBF\cdot {\left[H\right]}_{\alpha }$$

in which [H]α is the oxygenated heme molar concentration in the arteriole expressed in µmol/mL. In this study, an oxygenated heme molar concentration of 7.377 µmol/mL was used in accordance with research by Zhang and colleagues (Figure 1) [46]. Difference maps for CVR and CMRO_2_ were created by subtracting the follow-up (T1) MRI map from the baseline (T0) MRI map (Figure 1—Difference maps). Hereby positive values represent an increase in either CVR or CMRO_2_ at follow-up (T0 < T1) and negative values represent a decrease nown either CVR or CMRO_2_ at follow-up (T0 > T1). Lastly, based on the previously created edema masks, new masks were generated (Figure 1) to incorporate regions only exhibiting edema at baseline (edema T0), regions only exhibiting edema at follow-up (edema T1), and regions exhibiting edema at both time points (edema T0 + T1). The GTV (i.e., brain metastases) were excluded from all edema masks. Next, masks were created that incorporated healthy-appearing brain tissue at both time points. Therefore, the edema mask (edema T0 + T1) and PTV were extracted from the whole-brain tissue to make sure any possible malignant tissue was excluded. Then the healthy-appearing brain tissue was subdivided into grey matter (GM) and white matter (WM).

#### 2.4.2. Statistical Analysis

First, we compared the differences in the physiological MRI parameters between various tissue types before SRS. For each physiological MRI map, we calculated the average value per tissue type (GM, WM, edema and brain metastases) prior to SRS for each patient separately. We used Wilcoxon signed-rank tests to compare the average values across the different tissue types for each physiological MRI map. Secondly, we investigated whether there was a relationship between the extent of edema and the physiological MRI parameters within healthy-appearing brain tissue before SRS. Therefore, we computed the average value within the healthy-appearing brain tissue for each patient and then used Spearman’s rank correlations to assess the correlation between this value and the volume of edema (expressed as a percentage of voxels contained within the (extracted) whole brain mask).

Post-radiotherapy changes in the physiological MRI parameters were assessed separately for edematous tissue regions and healthy-appearing tissue. Regarding the edematous regions, we divided them into three distinct categories based on their temporal occurrence: (1) regions with edema at either T0 or T1 (i.e., all edema), (2) regions with edema exclusively at T1 (i.e., new edema), and (3) regions with edema exclusively at T0 (i.e., old edema; Figure 1). To evaluate the differences between the pre- and post-radiotherapy values for each type of edema and each physiological MRI map, we used Wilcoxon signed-rank tests. For healthy-appearing brain tissue, we calculated the mean values of each physiological parameter separately for the pre- and post-radiotherapy MRI scans, for each patient. Subsequently, we used Wilcoxon signed-rank tests to determine whether any changes occurred in the healthy-appearing brain tissue after SRS, by comparing the mean pre-radiotherapy values to the mean post-radiotherapy values.

Dose-related changes in healthy-appearing brain tissue were assessed using three different methods. First, we calculated the mean value for each physiological parameter map for each dose ROI (i.e., low, medium, high). Next, Wilcoxon signed-rank tests were performed to compare each of the ROIs to assess whether there was a significant difference between regions receiving either low, medium or high doses. For the second method, the radiotherapy dose map was sorted based on ascending values. The sorted dose values were then subdivided into 20 bins, each containing 5 percent of the data. Using the boundaries of these ‘5%-bins’, the dose map was divided into ROIs. Each ROI contained 5 percent of the dose data, where the lowest bin value represented the 5 percent lowest dose values and the highest bin value the 5 percent highest dose values. For each physiological MRI difference map, the mean value for each of these binned ROIs was calculated and used in the subsequent correlation analysis to assess whether the mean change in physiological MRI parameters in each bin-ROI correlated with the mean dose received by the tissue within that bin. As this binning results in 20 mean values per individual (i.e., for each binned ROI), a repeated measures correlation was chosen to account for the within-individual variance between these values. The ‘rmcorr’ package [47] implemented in rStudio (Version 2021.9.1.372) [48] was used to perform this repeated measures correlation. This analysis estimates the common regression slope for repeated measures. In addition to the two regional dose analyses, voxel-wise Spearman’s rank correlations were calculated between the delivered dose and physiological MRI difference maps for each patient separately. We performed Wilcoxon signed-rank tests to assess whether the correlation values significantly differed from zero on the group level for each physiological MRI parameter. For all statistical tests, a *p*-value < 0.05 was considered statistically significant, corrected for multiple comparisons when necessary.

## 3. Results

### 3.1. Participants

**Figure A2:**
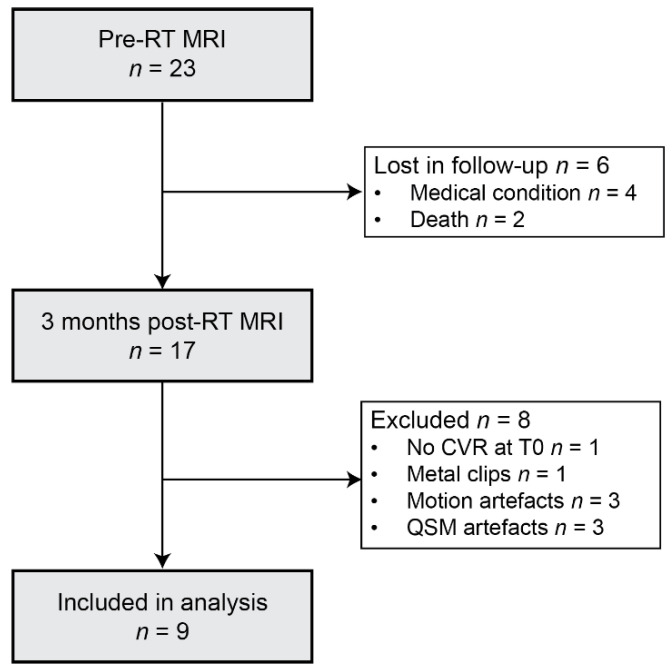
Flow-chart of patients included in the analysis for the current analysis.

In total, 17 patients completed both the baseline and follow-up MRIs and NCAs within the given time period. Of these, nine patients (four females) were included for the current analysis (Table 1). Most patients were excluded from the analysis due to artefacts in the MRI data (Figure A2). Three of the nine patients did not want to perform the breathing challenges during the BOLD imaging at follow-up, leading to missing CVR data for those patients.

All nine patients received SRS on a linear accelerator (Elekta) with conebeam CT imaging guidance, most commonly 21 Gy delivered in one fraction. The median age of the patients was 66 years and most patients had a primary lung tumor (all non-small cell lung cancers). The median number of brain metastases was seven (IQR 2-10) and the median total volume was 4 cc. Before SRS, the median edema volume excluding BM volumes was 17 cc and three months after SRS 6 cc, with extensive inter-individual variability. Most patients (6/9) had received systemic therapy during the follow-up period. The responses to SRS were evaluated approximately three months after treatment completion and the majority (4/9) showed decreased brain metastases volumes on follow-up scans, while 2/9 showed growth of new brain metastases. One patient had a mixed response, indicating some brain metastases had grown and others had shrunk.

### 3.2. Baseline Tissue Comparison

**Figure 2:**
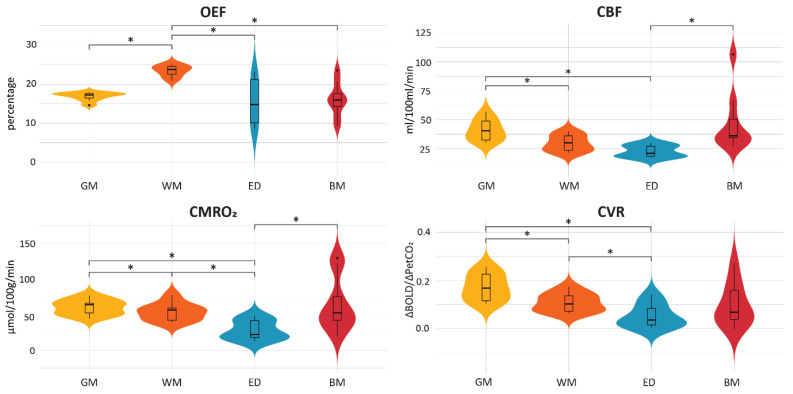
Violin plots displaying the differences in OEF, CBF, CMRO_2_ and CVR values across different tissue types. Asterisks indicate significant differences using a corrected *p*-value = 0.017 for multiple comparisons.

**Figure A3:**
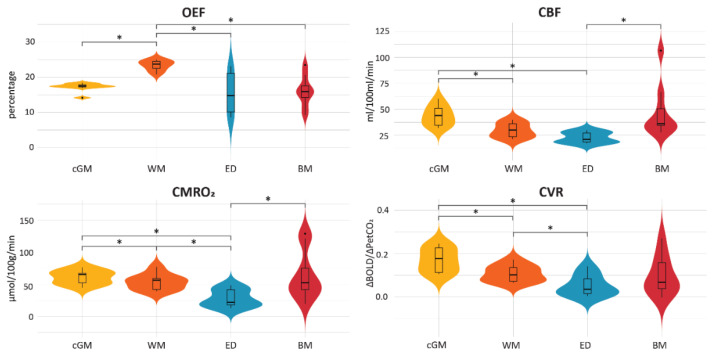
Violin plots displaying the differences in OEF, CBF, CMRO_2_ and CVR values across different tissue types. Asterisks indicate significant differences. Corrected *p*-value = 0.017. Abbreviations: BM, brain metastases; CBF, cerebral blood flow; cGM, cortical grey matter; CMRO_2_, cerebral metabolic rate of oxygen; CVR, cerebrovascular reactivity; ED, edema; OEF, oxygen extraction fraction; WM, white matter.

For each physiological MRI parameter, the group mean values were compared between different tissue (GM, WM, edema, brain metastases) using Wilcoxon signed-rank tests (Figure 2 and Table A2). A subset of these parameters has been reported previously [14]. In the current analyses, the GM had a higher CBF and CVR than both WM and edema (all *p* = 0.004). The WM CVR was higher than the CVR in edema regions (*p* = 0.008). For CMRO_2_, the GM values were higher than both WM and edema (*p* = 0.008 and *p* = 0.004, respectively), and the WM had higher CMRO_2_ values than edema (*p* = 0.008). For OEF, the GM values were lower than WM (*p* = 0.004), and the WM OEF values were higher than edema (*p* = 0.004). The brain metastases had lower OEF than in WM (*p* = 0.004) and higher CBF and CMRO_2_ than the edema regions (both *p* = 0.004). Excluding subcortical GM areas from the analyses did not lead to changes in these significant differences between tissues (Figure A3).

### 3.3. Edema Regional Comparisons

#### 3.3.1. Edema Volume Pre-Radiotherapy

**Figure 3:**
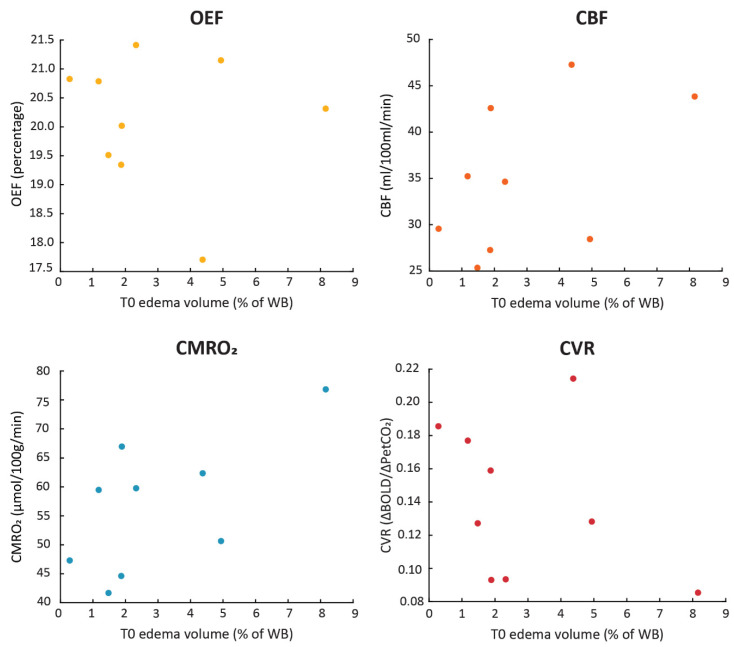
Relationship between the volume of edema pre-radiotherapy and the average physiological MRI parameters in the healthy-appearing brain tissue.

The relationship between the volume of edema (expressed as a percentage of the total brain volume) and the average physiological parameters within the healthy-appearing brain tissue pre-radiotherapy was calculated using Spearman’s rank correlations for each MRI metric (Figure 3). There were no significant correlations between the pre-radiotherapy edema volumes and the average whole-brain OEF (*r* = 0.02, *p* = 0.982), CBF (*r* = 0.43, *p* = 0.250), CMRO_2_ (*r* = 0.62, *p* = 0.086) or CVR (*r* = −0.45, *p* = 0.223).

#### 3.3.2. Post-Radiotherapy Changes

**Figure 4:**
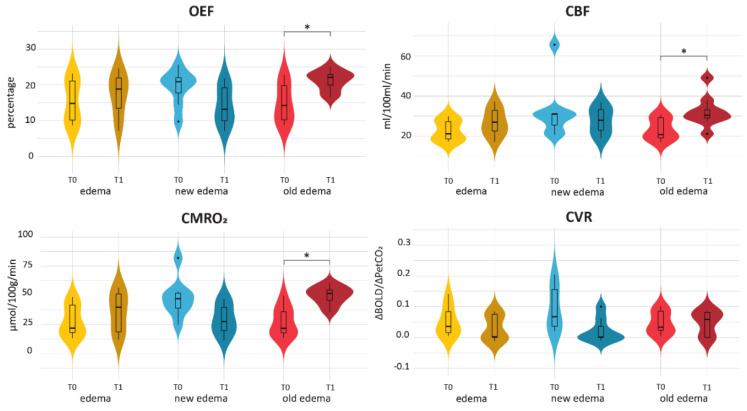
Violin plots of the pre- and post-radiotherapy values within different edema regions. Edema regions encompass any region with edema either pre- or post-radiotherapy. New edema indicates those areas with edema exclusively at post-radiotherapy and old edema indicates those areas with edema exclusively at pre-radiotherapy. Asterisks indicate significant differences.

To assess the influence of the presence of edema on the assessed MRI metric, the average values within the edematous regions were compared before and after SRS (Figure 4). Within edematous tissue, a distinction was made between regions with edema at both T0 and T1 (i.e., edema), regions with edema exclusively at T1 (i.e., new edema) and regions with edema exclusively at T0 (i.e., old edema). Comparisons were made using Wilcoxon signed-rank tests. The OEF, CBF and CMRO_2_ values in the old edema (T0 edema) regions were higher post-radiotherapy than pre-radiotherapy (all *p* ≤ 0.04), while the CVR values did not change in old edema (T0 edema) regions over time. When the outliers as visible in Figure 4 were removed from the analyses, the CBF values in the old edema regions (T0 edema) were no longer statistically significantly higher post-radiotherapy than pre-radiotherapy (*p* = 0.078). For OEF and CMRO_2_, the pre-post radiotherapy differences in old edema (T0 edema) remained statistically significant after outlier removal.

### 3.4. Healthy-Appearing Brain Tissue

#### 3.4.1. Global Post-Radiotherapy Changes

**Figure 5:**
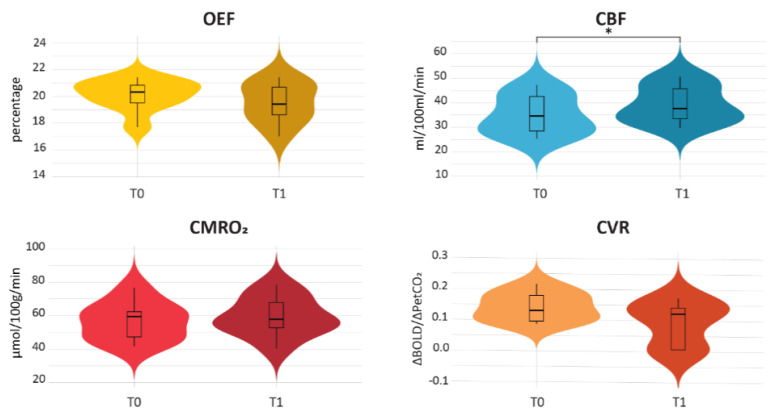
Comparison between global pre- and post-radiotherapy values within healthy-appearing brain tissue. Asterisks indicate significant differences.

To assess whether there were global effects of the radiotherapy on the healthy-appearing brain tissue, average MRI values were compared pre- and post-radiotherapy using Wilcoxon signed-rank tests (Figure 5). Only the CBF values were significantly higher post-radiotherapy than pre-radiotherapy (*p* = 0.02). This indicates that perfusion throughout healthy-appearing brain tissue increased after radiotherapy. None of the other MRI metrics showed significant changes after radiotherapy.

#### 3.4.2. Dose-Related Changes

**Figure 6:**
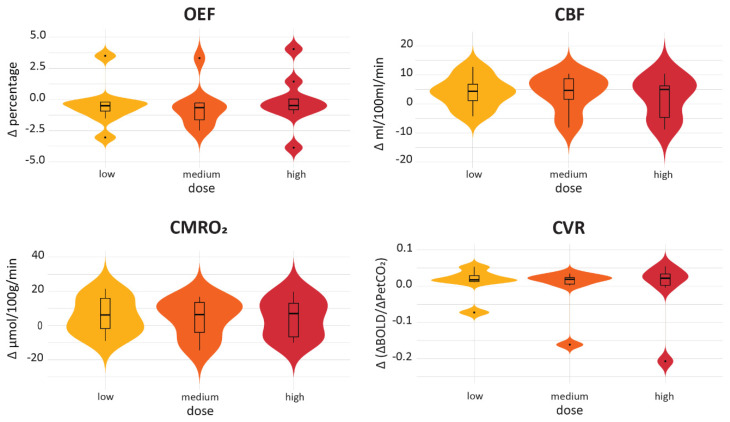
Comparison between the post-radiotherapy changes in the three dose ROIs within healthy-appearing brain tissue. Corrected *p*-value = 0.025.

Changes in the MRI metrics from pre- to post-radiotherapy were compared between healthy-appearing brain regions that had received low, medium and high radiotherapy doses (Figure 6). Differences between the dose groups were assessed using Wilcoxon signed-rank tests, which indicated none of the dose regions significantly differed from each other for any of the MRI metrics.

**Figure 7:**
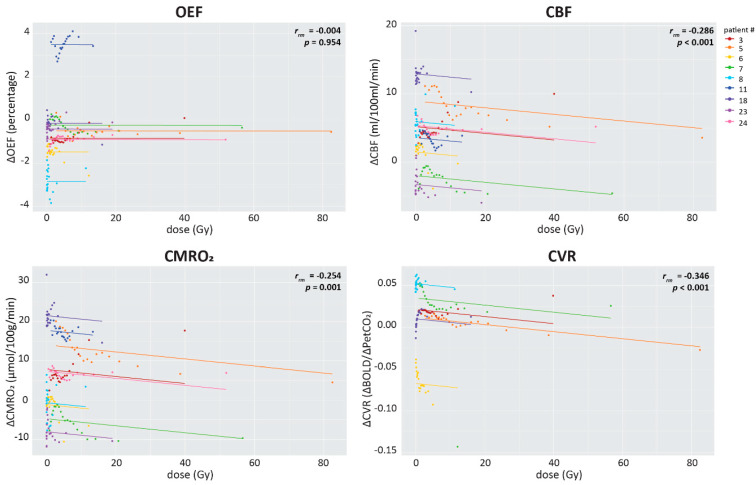
Repeated measures correlations of the post-radiotherapy changes and the dose in the healthy-appearing brain. Each color represents a different patient and the lines represent the common linear relationship between the MRI metrics when all participants are taken into account. Statistics for each relationship are provided for each plot separately.

**Figure A4:**
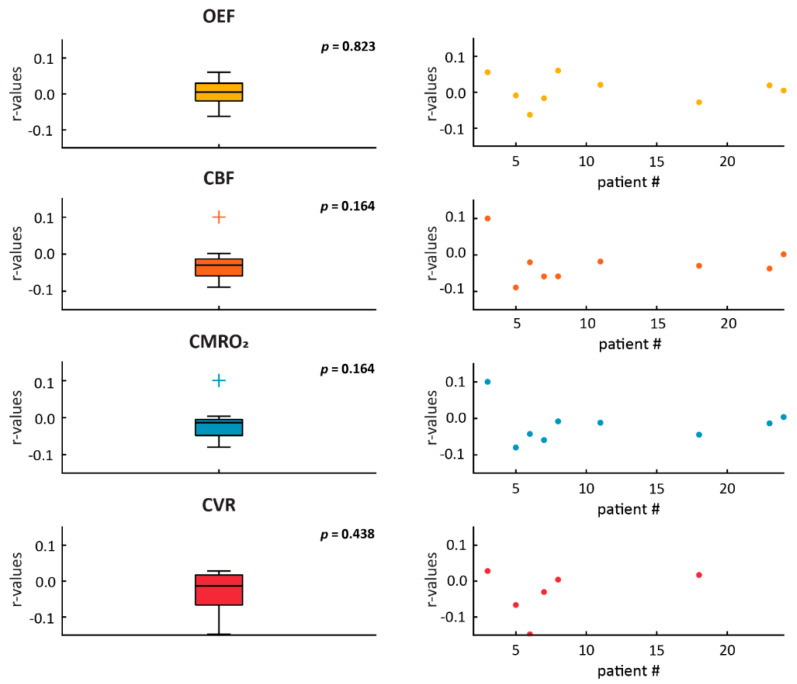
Voxel-wise correlation values for the association between delivered dose and post-radiotherapy change in OEF, CBF, CMRO_2_ and CVR in healthy-appearing brain tissue. Left column shows box-plots of the correlation values. *p*-values of the one-sided Wilcoxon signed-rank test are shown in the upper corner. Left column shows the correlation values for each individual patient. Abbreviations: CBF, cerebral blood flow; CMRO_2_, cerebral metabolic rate of oxygen; CVR, cerebrovascular reactivity; OEF, oxygen extraction fraction.

In addition to the ROI dose comparison, repeated measures correlations were performed between the dose and the change in each MRI metric post-radiotherapy using the dose-binned ROIs (Figure 7). The changes in OEF post-radiotherapy were not related to the delivered radiotherapy dose (*r_rm_* = −0.004, *p* = 0.954). There was a significant negative relationship between the delivered dose and the post-radiotherapy changes in healthy-appearing brain tissue regarding CBF (*r_rm_* = −0.286, *p* < 0.001), CMRO_2_ (*r_rm_* = −0.254, *p* = 0.001) and CVR (*r_rm_* = −0.346, *p* < 0.001). This indicates that post-radiotherapy declines in CBF, CMRO_2_ and CVR were related to higher delivered radiotherapy doses. Additionally, the voxel-wise Spearman’s rank correlations between the delivered radiotherapy dose and post-radiotherapy changes in OEF, CBF, CMRO_2_ and CVR were performed for each individual (see Figure A4). One-sample Wilcoxon signed-rank tests indicated that the correlation values did not significantly differ from zero on the group-level (all *p* > 0.05).

### 3.5. Case Analysis

**Figure 8:**
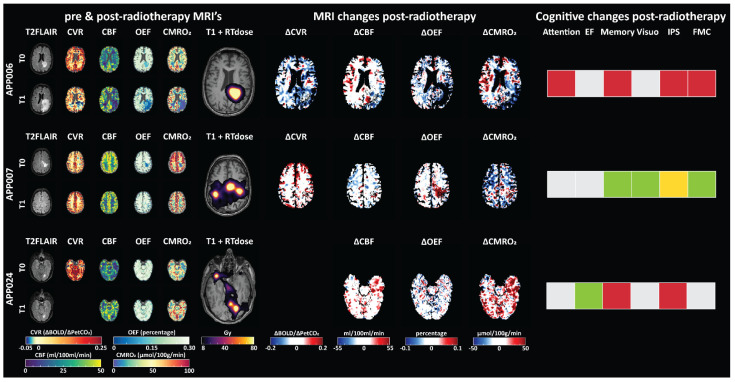
Visual comparison of MRI-related and cognitive changes in three subjects with different radiotherapy responses. Subject 006 had new brain metastases, subject 007 had brain metastases shrinkage and subject 024 had a mixed response whereby some brain metastases grew and some shrunk. Pre- and post-radiotherapy MRI maps are shown separately for pre-radiotherapy (T0) and three months post-radiotherapy (T1). Additionally, the delivered radiotherapy dose and difference scores (T1-T0) for the MRI maps are shown alongside the cognitive changes that were observed across the cognitive domains. Hereby grey indicates stable performance, green improvement, red decline and yellow mixed response. As patient 24 did not perform the CVR assessment post-radiotherapy, no difference maps for CVR are available. Abbreviations: EF, executive functioning; FMC, fine motor coordination; IPS, information processing speed; Visuo, visuoperception.

**Figure A5:**
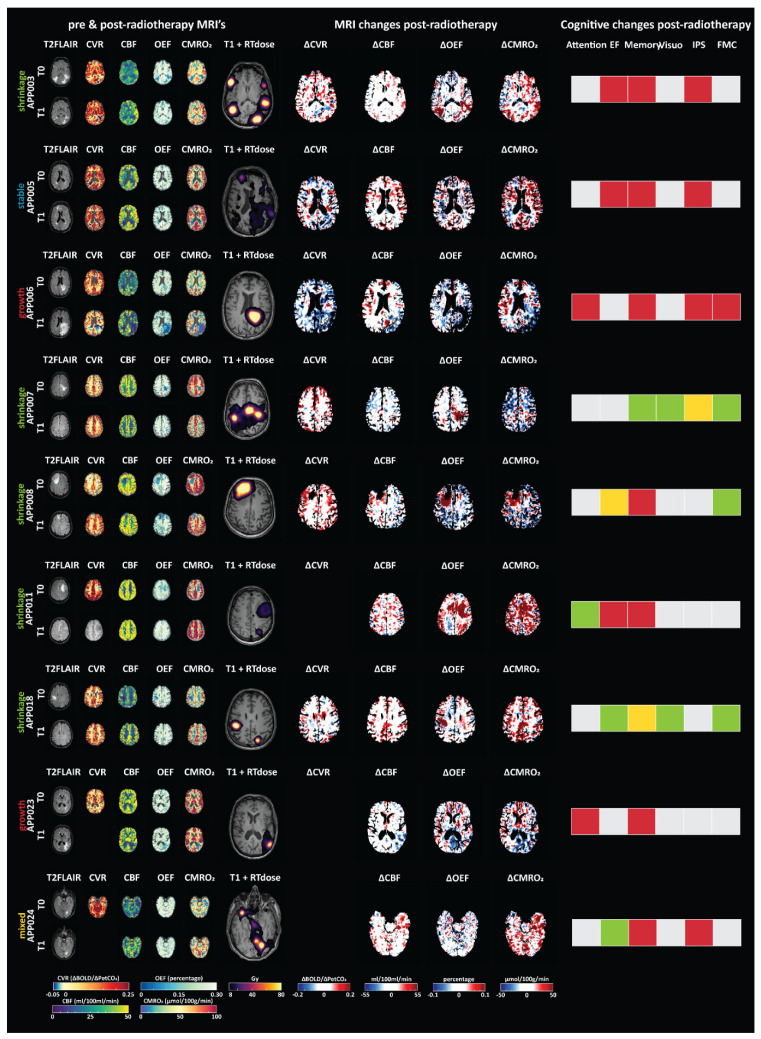
Visual comparison of MRI-related and cognitive changes in all subjects. Pre- and post-radiotherapy MRI maps are shown separately for pre-radiotherapy (T0) and three months post-radiotherapy (T1). Additionally, the delivered radiotherapy dose and difference scores (T1–T0) for the MRI maps are shown alongside the cognitive changes that were observed across the cognitive domains. Hereby grey indicates stable performance, green improvement, red decline and yellow mixed response. As some patients did not perform the CVR assessment post-radiotherapy, no difference maps for CVR are available for those patients. Abbreviations: CBF, cerebral blood flow; CMRO_2_, cerebral metabolic rate of oxygen; CVR, cerebrovascular reactivity; EF, executive functioning; FMC, fine motor coordination; IPS, information processing speed; OEF, oxygen extraction fraction; Visuo, visuoperception.

As group analysis indicates significant heterogeneity in the post-radiotherapy changes in OEF, CBF, CMRO_2_ and CVR, a case-study analysis was performed. Three diverse cases were chosen, based on their response to the radiotherapy: growth of new brain metastases, shrinkage of brain metastases and a mixed response three months after SRS (Figure 8). Besides changes in the physiological MRI parameter maps, the cognitive changes post-radiotherapy were assessed. For the patient that had new tumor growth after radiotherapy, the MRI differences and cognitive changes aligned; there was a general worsening of the physiological MRI parameters and cognitive decline in the majority of the cognitive domains. The largest changes in the physiological MRI values were near the tumor area. For the patient with tumor shrinkage, the improvement in the physiological MRI parameters was most pronounced for OEF where a post-radiotherapy increase of OEF was visible in the areas surrounding the tumor area. This improvement was also reflected in the cognitive changes, with improvement or stability in the majority of the cognitive domains. For the patient with a mixed response after radiotherapy, no clear pattern emerged from the MRI and cognitive data. See Figure A5 for a visual representation of all the included patients.

## 4. Discussion

In this study, we employed state-of-the-art physiological imaging techniques to comprehensively evaluate properties linked to both the metabolic and vascular reserve in patients with brain metastases before and three months after SRS. Our findings revealed a significant improvement in metabolic measures (OEF and CMRO_2_) within brain tissue that recovered from edema three months post-radiotherapy, while the vascular reserve remained impacted. Additionally, we observed a global post-radiotherapy increase in CBF in healthy-appearing brain tissue. No dose-related changes in any of the MRI metrics were detected when regions were categorized into low, medium and high-delivered doses. Correlation analyses highlighted larger reductions in CBF, CMRO_2_ and CVR within regions exposed to higher radiotherapy doses, but also indicated considerable variability both among patients and across dose regions. Case analyses suggested that part of this heterogeneity may be attributed to brain metastases progression following SRS. Collectively, our findings suggest that in this small patient sample no large metabolic or vascular changes occurred in the late-acute phase following SRS in patients with brain metastases.

Tissue-specific differences were observed pre-radiotherapy across both metabolic and vascular parameters. The tissue differences for CBF and CVR were similar to those reported in our previous study [14] and in healthy subjects [49], with both the WM areas and edema characterized by a lower CBF and CVR than GM. The CMRO_2_ values were higher in GM compared to WM, consistent with the higher neuronal density in GM and previous studies [50]. The OEF in GM was lower than in WM, while previous research has shown relatively homogeneous OEF in GM and WM using either QQ-CCTV [42] or PET [51]. Lower OEF values in deep GM structures due to iron depositions [52] cannot explain this phenomenon, as restricting the comparison to cortical GM yielded similar results. This may indicate the lower GM OEF is a compensatory auto-regulatory response to the increased GM CBF, aiming to sustain stable GM CMRO_2_. However, due to the smaller number of voxels present in the GM and thereby its heightened susceptibility to extreme values and minor spatial misalignment, this could also potentially account for some of the observed differences.

Large variability in the physiological parameters was seen within tissue containing untreated brain metastases, as was expected based on previous literature [12,14,53]. The primary tumor (i.e., origin of metastatic cells), might cause some of this heterogeneity as it affects the brain metastases growth pattern, metabolism and vasculature [6,10,54,55]. As the CMRO_2_ was not different from that of healthy-appearing tissue, this could be illustrative of the Warburg effect (i.e., less reliant on oxidative metabolism and more on aerobic glycolysis), especially since the OEF was lower than in healthy-appearing WM [56,57,58]. Moreover, we observed low OEF, CBF, CMRO_2_ and CVR within the edema regions. This is in line with previous research [13,14] and may be reflective of the local pressure in these regions restricting the ability of vessels to dilate and thus maintain adequate perfusion. However, the extent of the edema did not correlate to either the metabolic or vascular reserve in the healthy-appearing brain tissue. This indicates that the edema volume, which is indicative of the pressure exerted on the whole brain, does not appear to impact the overall vascular and metabolic capacities of the brain. Instead, its effects seem to be primarily localized. As the effect sizes of these non-significant relationships were moderate-to-high, future research should further validate these findings in larger samples. As patients with signs of increased intracranial pressure were excluded from participation, the generalizability of these findings to the wider brain metastases population may be limited.

We observed post-radiotherapy changes within the edematous regions. Notably, regions that recovered from edema three months after SRS showed increased OEF, CBF and CMRO_2_. Contrarily, CVR remained unchanged within these regions, with values lower than those observed in healthy-appearing tissue. These findings suggest that while these resolved edema regions show metabolic recuperation, the vascular reserve continues to be impacted. While there was a trend towards decreased OEF, CMRO_2_ and CVR in regions with new edema post-radiotherapy, statistical significance for CVR was almost reached (*p* = 0.078) after removing outliers. This lack of significance may be attributed to the limited number of patients available for analysis, emphasizing the need for further investigation. Altogether, our findings suggest that CVR might be more susceptible to, and serve as a sensitive marker of edema-induced effects, warranting continued exploration in future studies.

We observed a global increase in CBF throughout the brain after SRS, while other physiological measures remained constant. Studies have shown that exposure to radiation leads to an inflammatory response in the tissue [59,60]. The observed increase in CBF may thereby be either reflective of a restorative mechanism of the brain in response to radiation-induced inflammation or ongoing inflammation. However, as the majority of patients did not use dexamethasone three months after SRS, it is most likely indicative of a restorative mechanism. Previous studies have demonstrated that tissue exposed to a radiation dose exceeding 15 Gy suffers irreversible damage to its vasculature [17]. Surprisingly, our regional analysis did not reveal differences in post-radiotherapy changes among regions exposed to low (<10 Gy), medium (10–15 Gy) or high (>15 Gy) doses. However, it is important to consider that all patients in the current study received SRS, which effectively limits the radiation dose to the healthy brain tissue surrounding the tumor. This may have influenced our analysis, particularly in the high dose regions, where the number of voxels available for examination was considerably lower. In order to gain further insights, we therefore conducted regional analysis using equivalently sized ROIs (bins) that corresponded to increasing radiotherapy doses. Our analysis revealed a negative correlation between the dose and CBF, CMRO_2_ and CVR, but not OEF. This suggests that as the radiotherapy dose increases, the impact on both the metabolic and vascular capacities becomes more pronounced. While voxel-wise analyses did not support the associations observed in this ROI-based analyses, regional analysis is often more precise in evaluating these associations as demonstrated in previous research [14,61], due to the inherent measurement noise associated with these physiological MRI measures. As our current analyses focused solely on linear relationships, exploring non-linear relationships, especially those seen at low radiotherapy doses, would be valuable for future research. Further investigation is warranted regarding the potential vascular damage caused by the high fractional doses delivered during SRS, as previous studies have indicated that single fraction doses in particular may contribute to this effect [17].

Importantly, the analyses highlighted substantial variations both among patients and within dose regions. The case analyses suggested that part of this heterogeneity could be attributed to tumor progression following SRS. In our case study we examined three distinct cases with varying responses to SRS, including tumor growth, tumor shrinkage and a mixed response. In the case of new brain metastases growth, there was a deterioration in both physiological MRI parameters and cognitive function, particularly in areas near the tumor which also showed edema increases. Conversely, for the patient with tumor shrinkage, there was an improvement in physiological MRI parameters, specifically in OEF, which corresponded to positive changes in cognitive performance. In the case of a mixed response, less clear patterns emerged from the MRI and cognitive data with both improvements and declines. These results underscore the complex interplay between physiological changes, tumor response and cognitive outcomes, highlighting the need for individualized assessment and further research in this domain.

In this research we used state-of-the-art physiological imaging techniques in a heterogeneous patient population with the goal of identifying potential challenges and opportunities for future research. Due to the sensitivity of physiological MRI measures to acquisition artifacts, stringent patient selection was implemented, yet variations in the data could still arise from measurement and calculation discrepancies. For example, the choice of ASL technique can impact perfusion maps and subsequent CMRO_2_ calculations. An example is that too short post-labeling delays can lead to arterial transit artefacts or less accurate CBF measures [62,63,64]. We used a multi post-labeling delay ASL pcASL sequence and found CBF values that were close to the ground truth as measured using PET. The QQ-CCTV method used in this study has been validated against the reference standard 15O-PET with a good scan–rescan reproducibility [40] and demonstrated sensitivity for detecting physiological OEF changes, like the expected decreased OEF in hypercapnia compared to normoxia [65]. Also, QQ-CCTV has demonstrated OEF abnormalities in neurological disorders, including multiple sclerosis [66], ischemic stroke [46,67], brain tumor [68], dementia [69], pre-eclampsia [70], and hydrocephalus [71].

Several limitations should be considered when interpreting the findings of this study. In creating the QSM images, the precise positioning of the patients’ heads in the main magnetic field (B0) is crucial for accurate dipole inversion, which becomes challenging when scanning patients at multiple time points due to the inability to reproduce exact head positions. Thereby, subtle changes in QSM, and consequently OEF and CMRO_2_, may be attributed to variations in head positioning. Additionally, the biophysical model used to estimate OEF is not optimized for regions with edema. Specifically, the prolonged T2 relaxation time (~200 ms) observed in these regions exceeds the maximum echo-time of the ME-GRE sequence (44.5 ms) [72]. However, as CVR is also affected within edema regions, it is likely other autoregulatory mechanisms, including OEF, play an important role. Furthermore, an inherent limitation of using BOLD-metrics is the dependency of the BOLD response on multiple factors, including changes in cerebral blood volume, the cerebral metabolic rate of oxygen consumption, the arterial partial pressure of oxygen, and baseline parameters such as hematocrit, OEF, CMRO_2_ and blood volume [73,74]. This complex interplay hinders the identification of precise underlying mechanisms, but also underscores the value of integrating multiple physiological MRI measures. Lastly, compared to 7T scanners, the BOLD and ASL signal sensitivity, especially in WM and edema, is notably lower. Combined with the heterogeneous and small patient sample in this preliminary study, a lack of significant findings could partly be attributed to these factors.

## 5. Conclusions

Our findings suggest that resolved edema regions show metabolic recuperation but ongoing vascular damage, highlighting the sensitivity of CVR as a marker for vascular changes. We also observed a global increase in CBF in healthy-appearing brain regions following SRS, possibly indicating a restorative mechanism against radiation-induced inflammation. While regions exposed to higher doses exhibited larger declines in CBF, CMRO_2_ and CVR, there was notable heterogeneity among patients and across dose regions. The case analysis demonstrated some of this heterogeneity may be attributed to the tumor response. Overall, our preliminary results suggest that within the 3-month follow-up window no radiotherapy effects on physiological parameters occurred in healthy-appearing brain tissue. Nevertheless, while SRS can lead to local control of the brain metastases it can also have long-term side-effects (i.e., months to years) [75,76]. Continued long-term research with larger patient samples allowing for meaningful grouping of patients will enhance our understanding of the intricate interplay between radiation dose, brain health and physiological responses.

## Figures and Tables

**Table 1:** Patient, treatment and volume information of the included subjects in this study.

#	Age (Years)	Sex	Primary Tumor	#BMs	Total BMs vol (cc)	Prescribed SRS Dose (Gy/1 fr)	Previous RT	In between RT	Post-RT Systemic Therapy	Edema vol T0 (cc)	Edema vol T1 (cc)	Dexamethasone Dose Pre-RT (mg/Day)	Dexamethasone Dose Post-RT (mg/Day)	RT Response ^a^
3	57	F	Lung	7	4.3	21	N	N	N	54.3	21.4	0.0	1.5	Decrease
5	66	M	Melanoma	18	0.9	21	Y	N	Y	12.5	98.8	0.0	0.0	Stable
6	81	M	Melanoma	2	7.1	21	N	N	Y	17.1	155.3	0.0	0.5	Growth
7	62	F	Lung	11	11.2	21	N	N	Y	19.5	1.4	0.0	0.0	Decrease
8	72	M	Kidney	1	17.6	18	N	N	Y	80.1	5.7	4.0	0.0	Decrease
11 ^b^	52	F	Lung	8	50.6	15	N	N	Y	29.4	4.7	4.0	0.0	Decrease
18	72	M	Lung	2	1.8	21	N	N	N	13.2	0.4	2.0	0.0	Decrease
23 ^b^	74	F	Lung	1	3.5	18	N	Y	N	16.3	56.4	0.5	2.5	Growth
24 ^b^	62	M	Melanoma	10	1.6	24	N	N	Y	3.1	4.5	0.0	0.0	Mixed

^a^ based on the radiology report of the clinical scan; ^b^ no post-radiotherapy CVR maps available. Abbreviations: BMs, brain metastases; F, female; M, male; N, no; RT, radiotherapy; T0, pre-radiotherapy, T1, post-radiotherapy; vol; volume; Y, yes.

## Data Availability

Upon completion of the APRICOT trial, data may be made available pending a formal research proposal. The BOLD analysis scripts are openly available via the SeeVR toolbox (https://www.seevr.nl/ accessed on 26 April 2022). The ASL CBF analysis scripts are openly available via the ClinicalASL toolbox (https://github.com/JSIERO/ClinicalASL accessed on 29 June 2023).

## Abbreviations

The following abbreviations are used in this manuscript:

**ASL**, arterial spin labeling; **APRICOT**, Assessing and Predicting Radiation Influence on Cognitive Outcome using the cerebrovascular stress Test; **BOLD**, blood oxygen level dependent; **CBF**, cerebral blood flow; **CMRO_2_**: cerebral metabolic rate of oxygen; **CSF**, cerebrospinal fluid; **CVR**, cerebrovascular reactivity; **EF**, executive functioning; **EQD2**, equivalent total doses per 2-Gy fractions; **FMC**, fine motor coordination; **(f)MRI**, (functional) magnetic resonance imaging; **GM**, grey matter; **GTV**, gross tumor volume; **Gy**, gray; **IPS**, information processing speed; **MEDI**, morphology enabled dipole inversion; **ME-GRE**, multi-echo gradient-recalled-echo (ME-GRE); **NCA**, neurocognitive assessment; **PetCO_2_**, end-tidal CO_2_; **PDF**, projection onto dipole field; **PLD**, post label delay; **PTV**, planned target volume; **QQ**, QSM + qBOLD; **QQ-CCTV**, QQ-based combined cluster analysis of time evolution and tissue type with total variation denoising; **SEPIA**, Susceptibility mapping pipeline tool for phase images; **OEF**, oxygen extraction fraction; **ROI**, region of interest; **T1FFE**, three-dimensional T1 weighted fast field echo; **Visuo**, visuoperception; **WM**, white matter.

## Appendix A. In- and Exclusion Criteria

Inclusion criteria:

- Age ≥ 18 years;
- Either radiographic and/or histologic proof of metastatic brain disease eligible for brain radiation therapy;
- Eligible for brain irradiation for prophylaxis or treatment;
- Expected survival ≥ 5 months, as determined by graded prognostic assessment (GPA) score;
- Sufficient knowledge of the Dutch language to allow reliable use of the standardized tests and understand the study information;
- Participation in the COIMBRA cohort, with given consent for filling in quality of life questionnaires.

Exclusion criteria:

- Standard contraindications for 3T MRI scanning;
- Standard contraindications for using the RespirAct RA-MRTM MRI UNIT (see D2 IMDD 2.5 contraindications);
- Medical contraindications to limited hypercapnia (known metabolic acidosis or alkalosis);
- Unwilling or unable to cooperate with breathing maneuvers or keeping still;
- Noncompliance with prescribed anti-seizure medication;
- Severe current neurological or psychiatric diseases not related to the primary malignancy or cerebral metastases;
- History of cerebrovascular disease (ischemic stroke or intracranial hemorrhage);
- Non-prophylactic use of >4 mg dexamethasone on the day of participation;
- Cardiovascular disease: congestive heart failure (New York Heart Association Class III to IV), symptomatic ischemia, conduction abnormalities uncontrolled by conventional intervention or myocardial infarction within past 6 months;
- Pulmonary disease: oxygen dependency at rest or with exercise, restrictive lung disease with resting respiratory rate over 15 breaths/min;
- Concurrent severe or uncontrolled medical disease (e.g., active systemic infection);
- History of bleomycin treatment;
- Body weight < 30 kg or >100 kg;
- Pregnancy.

## Appendix B. Neuropsychological Tests

**Table A1:** Neuropsychological tests per neurocognitive domain.

**Attention**
Wechsler Adult Intelligence Scale (WAIS-IV), Digit Span Forward Score
Trail Making Test (TMT), switching ratio B/A
Stroop/Delis Kaplan Executive Function System (DKEFS), switching ratio IV vs. III
**Executive functioning**
WAIS-IV, Digit Span Backward Score
Letter fluency (3 letters)
Stroop D’KEFS, inhibition ratio III vs. I
**Memory**
Hopkins Verbal Learning Test—Revised (HVLT-R), immediate, delayed and recognition
Rey–Osterieth Complex Figure Test (ROCFT), delayed copy
Visual Association Test (VAT), long form, immediate and delayed
Semantic fluency
**Processing speed**
TMT A
Stroop/DKEFS, naming [I] and reading [II]
Psychomotor speed
Lafayette Grooved Pegboard, dominant and non-dominant hand
**Visuospatial functioning**
ROCFT, direct copy
Hooper Visual Organization Test (HVOT), fragmented
**Social cognition**
Facial Expressions of Emotion—Stimuli and Tests (FEEST), total score
**Language**
HVOT, non-fragmented [clinical interpretation]

## Appendix C. Numerical Baseline Physiological Values

**Table A2:** Group mean values (standard deviation) per MRI metric (OEF, CBF, CMRO_2_, CVR) per tissue type (WB, GM, WM, edema and brain metastases).

MRI Metric	GM	WM	Edema	Brain Metastases
OEF (percentage)	16.9 (1.1)	23.4 (1.5)	15.5 (6.1)	16.0 (4.3)
CBF (mL/100 mL/min)	41.0 (9.1)	29.6 (7.0)	22.8 (5.2)	46.8 (25.5)
CMRO_2_ (µmol/100 g/min)	60.1 (10.7)	53.7 (12.3)	27.9 (13.4)	63.6 (38.1)
CVR (∆BOLD/∆PetCO_2_)	0.17 (0.06)	0.11 (0.04)	0.05 (0.05)	0.10 (0.09)

Abbreviations: GM, grey matter; WM, white matter.
